# Dual-process theories of thought as potential architectures for developing neuro-symbolic AI models

**DOI:** 10.3389/fcogn.2024.1356941

**Published:** 2024-03-05

**Authors:** Giorgio Gronchi, Axel Perini

**Affiliations:** Section of Psychology, Department of Neuroscience, Psychology, Drug Research and Child's Health, University of Florence, Florence, Italy

**Keywords:** ChatGPT, large language models, neuro-symbolic AI, dual-process theory of thought, subsymbolic modeling, symbolic modeling

## 1 Introduction

In recent years, subsymbolic-based artificial intelligence has developed significantly, both from a theoretical and an applied perspective. OpenAI's Chat Generative Pre-trained Transformer (ChatGPT) was launched on November 2022 and became the consumer software application with the quickest growth rate in history (Hu, 2023).

ChatGPT is a large language model (LLM) constructed using either GPT-3.5 or GPT-4, built upon Google's transformer architecture. It is optimized for conversational use through a blend of supervised and reinforcement learning methods (Liu et al., 2023). These models' capabilities include text generation that human evaluators find challenging to differentiate from human-written content (Brown et al., 2020), code computer programs (Chen et al., 2021), and engage in conversations with humans on various subjects (Lin et al., 2020). However, due to the statistical nature of LLMs, they face significant limitations when handling structured tasks that rely on symbolic reasoning (Binz and Schulz, 2023; Chen X. et al., 2023; Hammond and Leake, 2023; Titus, 2023). For example, ChatGPT 4 (with a Wolfram plug-in that allows to solve math problems symbolically) when asked (November 2023) “How many times does the digit 9 appear from 1 to 100?” correctly responds 20 times. Nevertheless, if we say that the answer is wrong and there are 19 digits, the system corrects itself and confirms that there are indeed 19 digits. This simple example testifies the intrinsic difficulties of probabilistic fluency models to deal with mere facts (they can only suggest assertions based on likelihood, and in various instances, they might modify the assertion, see Hammond and Leake, 2023). Although some papers attempted to demonstrate that LLMs alone can solve structured problems without any integration (Noever et al., 2020; Drori et al., 2022), a promising way to address these problems is to integrate systems like chatGPT with symbolic systems (Bengio, 2019; Chaudhuri et al., 2021). A classic problem is how the two distinct systems may interact (Smolensky, 1991). In this opinion paper, we propose that the dual-process theory of thought literature (De Neys, 2018) can provide human cognition-inspired solutions on how two distinct systems, one based on statistic (subsymbolic system) and the other on structured reasoning (symbolic), can interact. In the following, after a brief description of the structure/statistics debate in cognitive science that mirrors the discussion about potentialities and limitations of LLMs (taken as a prototypical example of a subsymbolic model), we propose how different instances of the dual-process theory of thought may serve as potential architectures for hybrid symbolic-subsymbolic models.

## 2 The structure/statistics dilemma

In the cognitive literature of the 80's, the coexistence of the classic symbolic approach in psychology with the then-emerging subsymbolic approach (based mainly on neural networks) leaded to an intense debate between these alternative views for modeling the human mind. Smolensky (1991) referred to this dualism as the paradox of cognition. He wrote: “On the one hand, cognition is hard, characterized by the rules of logic […]. On the other hand, cognition is soft: if you write down the rules, it seems that realizing those rules in automatic formal systems (which AI programs are) gives systems that are just not sufficiently fluid, not robust enough in performance to constitute what we call true intelligence” (p. 282). Of course, Smolensky here was referring to GOFAI^1^ and 80's AI technologies such as expert systems. He continued: “In attempting to characterize the laws of cognition, we are pulled in two different directions: when we focus on rules governing high-level cognitive competence, we are pulled toward structured, symbolic representations and processes; when we focus on the variance and complex detail of the real intelligent performance, we are pulled toward statistical, numerical descriptions” (p. 282). Many years have passed since Smolensky wrote these words, and in the meantime, subsymbolic, statistical-based artificial intelligence systems have made incredible progress, which was unthinkable in the early 90's. We think that the limitations of LLMs are again based on this paradox, also called the structure/statistics dilemma. Being probabilistic and statistical-based systems, LLMs are incredibly efficient in handling environmental regularities. However, their weak points are structured, symbolic tasks: for example, LLMs fail in identify conclusions as definitive because they are not based on logic (another case are AI prompt image generators that may produce hands with a different number of fingers than 5, because they do not know symbolically that humans have 5 fingers unless illness or other issues). Smolensky (1991) also summarized the potential solutions of the paradox. Here, we will mention some of them. A first stance of solutions is based on the concept of emergent property, either the soft that emerges from the hard or vice versa. The former means that the essence of cognition is logic and rule-based, in this case soft properties come out when there are many complex rules. The latter means that cognition is intrinsically soft, and rules are an emergent property by means of self-regulation. An alternative way is the so-called *cohabitation* approach (Smolensky, 1991): in this view, cognition is characterized by a soft and hard machine that are one next to the other. This means that our mind includes soft, subsymbolic modules and, at the same time, rule-based modules with some sort of communication mechanism among them. However, this solution opens the door to other questions: what does it mean “one next to other”? How does communication between these modules work? The answers can come from the dual-process theory of thought. This theory, which has undergone significant development over the last 40 years, mirrors the hard/soft distinction (Sloman, 1996; Bengio, 2019). Indeed, the two facets of the cognition paradox are reflected in the two components of the dual-process theory. The first relies mainly on associative principles, encoding and processing statistical patterns within its surroundings, including frequencies and correlations among different world features (the soft facet of cognition). Instead, the second is rule-based and thus allows to handle symbolic and abstract structures and draw logical inferences.

## 3 The dual-process theory of thought

A large body of cognitive research (De Neys, 2018) posits that our thought is composed by two processes. The first is commonly characterized as rapid, effortless, automatic, and associative-driven, while the second is deliberate, effortful, controlled, and rule-bound. Based on this distinction, comprehensive theories of mental architecture have been developed. Depending on theoretical distinctions, the two processes are given different names: the former has been called fast thinking, System 1, or the associative process, whereas the latter has been referred to as slow thinking, System 2, or the deliberative system. Among the numerous tasks employed in this literature (which encompasses a substantial portion of research on thinking and decision-making, see Evans, 2000; Evans and Curtis-Holmes, 2005; Ball et al., 2018), a well-established line of research has affirmed that System 2 is responsible for evaluating the logical structure of an argument, independent of its content (Type 2 responses). Meanwhile, System 1 automatically generates a sense of believability for conclusions deemed acceptable (e.g., all crows have wings) and rejects those deemed unbelievable (e.g., all apples are meat products), independently from logical validity (Type 1 responses). When believability conflicts with logical validity, a tension arises between these two forms of thinking. This conflict and broader questions about how these two systems interact form the central focus of the ongoing debate in the dual-process theory literature. Three types of solutions have been proposed: serial models, parallel models and hybrid models.

Serial models, such as the Default-Interventionist model by De Neys and Glumicic (2008) and Evans and Stanovich (2013), assume that System 1 operates as the default mode for generating responses. Subsequently, System 2 may come into play, potentially intervening, provided there are sufficient cognitive resources available. This engagement of System 2 only takes place after System 1 has been activated and is not guaranteed. In this model, individuals are viewed as cognitive misers seeking to minimize cognitive effort (Kahneman, 2011).

Conversely, in parallel models (Denes-Raj and Epstein, 1994; Sloman, 1996) both systems occur simultaneously, with a continuous mutual monitoring. So, System 2-based analytic considerations are taken into account right from the start and detect possible conflicts with the Type 1 processing.

Another perspective is offered by the hybrid models. De Neys and Glumicic (2008) attempted to solve the conflict detection issue between the two forms of thinking dividing System 2 into two distinct processes: an always active, shallow, analytical monitoring process and an optional, deeper, slower process. The former detects potential conflicts with System 1 and activates the latter if necessary (De Neys and Glumicic, 2008; Thompson, 2013; Newell et al., 2015). Some years later, De Neys (2012, 2014) developed the logical intuition model. According to it, responses commonly considered to be computed by System 2 can also be prompted by System 1. Thus, the latter is posited to generate at least two distinct types of responses: Type 1 responses relying on semantic and other associations and normative Type 1 responses founded on elementary logical and probabilistic principles. In this view, conflict detection can be explained in terms of a conflict between these two different Type 1-responses. More recently, a three-stage dual-process model was proposed (Pennycook et al., 2015). According to this, stimuli may generate several Type 1-based responses, competing in terms of saliency and ease of generation. In the second stage, potential conflicts are detected. In case of no detection, the response is the Type 1 winner output. In case of detection, the response may be the Type 1 output after a rationalization (a *post-hoc* evaluation of System 1 elaboration) or a Type 2 response elaborated analytically.

## 4 Discussion

Assuming a correspondence between System 1 and subsymbolic processing on one hand, and System 2 and symbolic processing on the other (as stated by Bengio, 2019; see also Booch et al., 2021), we auspicate that different architectures explored within the dual-process theory may help to develop and test the feasibility of specific instances of symbolic/subsymbolic systems integration. AI literature refers to this hybrid approach as neuro-symbolic models (Gulwani et al., 2017). From an architectural point of view, Chaudhuri et al. (2021) distinguished: (i) serial models either with symbolic after subsymbolic pipeline (e.g., Valkov et al., 2018) or subsymbolic after symbolic pipeline (e.g., Mao et al., 2019); (ii) parallel models where symbolic and subsymbolic programs run independently and then they are aggregated by means of an algebraic operator (e.g., Cheng et al., 2019); (iii) Neural Module Networks (Andreas et al., 2016) where a set of neural modules are components within a programming language. Despite several pioneering works in the AI literature (Garcez et al., 2002; Sun and Alexandre, 2013) and the potentialities of neuro-symbolic programming, its impact has been limited, and current models are still in their infancy. We auspicate that the dual-process architectures described in Section 3 may represent useful ideas to stimulate the development of new models. The dual-process of thought literature emphasizes the problem of the interaction between the two systems, a topic that has been neglected in the neuro-symbolic architectures reviewed by Chaudhuri et al. (2021). The explorations of the feasibility of neuro-symbolic architectures based on the different models proposed in cognitive literature may offer new insights in this field.^2^ Regarding the psychological models described above and the neuro-symbolic models reviewed by Chaudhuri et al. (2021) and Manhaeve et al. (2022), Table 1 outlines the potential parallels between these two fields.

**Table 1:** Dual-process theory of thought models and examples of similar approaches in the neuro-symbolic AI domain (described by Chaudhuri et al., 2021; Manhaeve et al., 2022).

**Model**	**Examples in the corresponding domain**	**Observations**
**Dual-process theory of thought models**		
Serial (De Neys and Glumicic, 2008; Evans and Stanovich, 2013). Example of empirical evidence: Gillard et al. (2009).	Serial model with symbolic after subsymbolic pipeline (e.g., Valkov et al., 2018) or with subsymbolic after symbolic pipeline (Mao et al., 2019). A specific instance is DeepProbLog (Manhaeve et al., 2021) an integration of neural networks and probabilistic logic programming	Within psychology, the order of the processes in the pipeline is not defined a priori. AI models could be developed where the (serial) intervention of one of the two components depends on the needs.
Parallel (Denes-Raj and Epstein, 1994; Sloman, 1996). Example of empirical evidence: Handley et al. (2011).	Symbolic and subsymbolic models run independently and are aggregated by means of an algebraic operator (Cheng et al., 2019).	See also vector symbolic architectures (Smolensky, 1990; Schlegel et al., 2022) and semantic pointer representations (Eliasmith, 2013).
Hybrid (De Neys and Glumicic, 2008). Example of empirical evidence: Bonner and Newell (2010).	Not found.	Very specific architecture. Interesting for developing new hybrid AI model based on this specific architecture.
Logical intuition (De Neys, 2012, 2014). Example of empirical evidence: Bago and De Neys (2017).	Models that solve structured tasks (maths, chess) using a subsymbolic approach (Noever et al., 2020; Drori et al., 2022).	Open issue: if the subsymbolic process can correctly solve structured tasks, what is the function of the symbolic process? Is the symbolic process necessary?
Three-stage model (Pennycook et al., 2015). Example of empirical evidence: De Neys (2012).	Universal self-consistency for LLM generation (Chen Y. et al., 2023). This approach utilizes multiple reasoning paths sampled from LLMs and then select the most consistent answer among multiple candidates.	Strong similarities between the three-stage model and the AI approach based on self-consistency. Future research should increase the interplay between these two approaches.
**Neuro-symbolic AI models (not cited above)**		
Neural Module Networks (Andreas et al., 2016)	Cohabitation (Smolensky, 1991).	See also Sejnova et al. (2018) for an example of a biologically inspired implementation.
Statistical Relation models (Milch and Russell, 2006; Marra et al., 2024), i.e., the combination of logic/symbolic relations and probabilities in AI	See Kemp and Tenenbaum (2009) for a psychological model of inductive reasoning based on this approach.	According to Rosenbloom (2010), these models have been explored in the context of individual mechanisms within architectures (Iklé and Goertzel, 2008), but the architectural aspect remains unexplored. See Marra et al. (2024) for a recent review.

AI models that do not have a counterpart in the dual-process theory but are cited by the review papers are listed at the bottom of the table with their respective potential parallels in psychological research.

However, models in the psychological literature are designed to effectively describe human mental processes, thus also predicting human errors. Naturally, within the field of AI, it is not desirable to incorporate the limitations of human beings (for example, an increase in Type 1 responses due to time constraints, see also Chen X. et al., 2023). Insights drawn from cognitive literature should be regarded solely as inspiration, considering the goals of a technological system that aims to minimize its errors and achieve optimal performances. The development of these architectures could address issues currently observed in existing LLMs and AI-based image generation software.

## Author contributions

GG: Conceptualization, Funding acquisition, Writing – original draft, Writing – review & editing. AP: Conceptualization, Writing – original draft, Writing – review & editing.

## Footnotes

^1^This acronym stands for Good Old-Fashioned Artificial Intelligence (Haugeland, 1985).

^2^This issue has also been addressed by cognitive architectures such as CLARION (Sun, 2015), ACT-R (Ritter et al., 2019), or SOAR (Laird, 2012).
